# Systematic review: a review of adolescent behavior change interventions [BCI] and their effectiveness in HIV and AIDS prevention in sub-Saharan Africa

**DOI:** 10.1186/s12889-017-4729-2

**Published:** 2017-09-18

**Authors:** M. Mwale, A. S. Muula

**Affiliations:** 1 Department of Public Health, School of Public Health and Family Medicine, University of Malawi College of Medicine, Mzuzu University, Private Bag 201, Luwinga Mzuzu 2, Blantyre, Malawi; 2 Department of Public Health, School of Public Health and Family Medicine, University of Malawi College of Medicine, Blantyre, Malawi

**Keywords:** Adolescent, HIV and AIDS, BCI interventions, Peer education, Abstinence

## Abstract

**Background:**

Despite sub-Saharan Africa [SSA] constituting just 12% of the world’s population, the region has the highest burden of HIV with 70% of HIV infection in general and 80% of new infections among young people occuring in the region. Diverse intervention programmes have been implemented among young people but with minimal translation to behavior change. A systematic review of Behavior Change Interventions [BCI] targeting adolescents in SSA was therefore conducted with the objective of delineating this intervention vis-a-vis efficacy gap.

**Methods:**

From April to July 2015 searches were made from different journals online. Databases searched included MEDLINE, EBSCOhost, PsychINFO, Cochrane, and Google Scholar; Cambridge and Oxford journal websites, UNAIDS and WHO for studies published between 2000 and 2015. After excluding other studies by review of titles and then abstracts, the studies were reduced to 17. Three of these were randomized trials and five quasi-experimental. Overall interventions included those prescribing life skills, peer education [*n* = 6] and community collaborative programmes. The main study protocol was approved by the University of Malawi College of Medicine Ethics Committee on 30th June 2016 [ref #: P.01/16/1847. The review was registered with PROSPERO [NIH] in 2015.

**Results:**

The review yielded some 200 titles and abstracts, 20 full text articles were critically analysed and 17 articles reviewed reflecting a dearth in published studies in the area of psychosocial BCI interventions targeting adolescents in SSA. Results show that a number of reviewed interventions [*n* = 8] registered positive outcomes in both knowledge and sexual practices.

**Conclusions:**

The review demonstrates a paucity of psychosocial BCI studies targeting adolescents in SSA. There are however mixed findings about the effectiveness of psychosocial BCI targeting adolescents in SSA. Other studies portray intervention effectiveness and others limited efficacy. Peer education as an intervention stands out as being more effective than other psychosocial regimens, like life skills, in facilitating HIV risk reduction**.** There is therefore need for further research on interventions employing peer education to substantiate their potential efficacy in HIV risk reduction among adolescents.

**PROSPERO registration number:**

CRD42015019244, available from http://www.crd.york.ac.uk/PROSPERO/display_record.asp?ID=CRD42015019244.

**Electronic supplementary material:**

The online version of this article (10.1186/s12889-017-4729-2) contains supplementary material, which is available to authorized users.

## Background

The HIV and AIDS pandemic continues to pose a threat to and undermine global public health. The fact that a vaccine and cure for the disease are yet to be discovered continues to confound public health efforts and global biomedical as well as clinical research. In most parts of the world however the spread of HIV has been halted. There are also indications of trend reversal which furnishes hope but no cause for complacency. Sub-Saharan Africa is the epicenter of the global pandemic. The high incidence of HIV in this region has been widely documented [1–8]. Of the estimated more than 34.2 million people infected with HIV globally over three quarters are estimated to live in SSA representing about 70% of the global infection burden yet the region only accounts for about 12% of the global population [8]. There is a high rate of HIV and AIDS cases among young people aged 15–24 years globally with females being disproportionately affected. On average 2, 500 young people [15–24 years] get infected with HIV every day and 80% of these infections take place in sub-Saharan Africa [9]. Unfortunately, strategies and interventions aimed at preventing new infections among young people in sub-Saharan Africa seem not to be having a significant impact in most nations in the region.

A majority of interventions among adolescents in SSA have been school based BCI which according to Michielsen are aimed to promote actions such as: abstinence or delaying the onset of first sexual intercourse, increasing condom use and reducing the number of sexual partners [9]. Additionally these interventions aim to increase knowledge, change attitudes, improve access to services and to reduce stigma or address other mediators as self-esteem and self-efficacy. Michielsen quoting Coates, further reports that such behavioural strategies attempt to motivate behavioural change within individuals and social units by use of a range of educational, motivational, peer-group, skills-building approaches, and community normative approaches [9, 10].

Most such interventions in SSA have however not registered impressive impact after evaluation in terms of targeted outcome measures. Previous evaluations that have endeavored to investigate this gap have pointed to among other limitations: misaligned theoretical basis [6, 11–13]; targeting the wrong age group especially older instead of younger adolescents who are not yet sexually active. Programmes elsewhere have provided evidence that it is easier to establish low-risk behaviour than to change existing behaviours resulting in calls to target younger adolescents or at the minimum tailor interventions to adolescent age categories [14–17]. Other limitations pointed to include, narrow focus on individual determinants of sexual behaviour [9]; implementation bottlenecks [18]; and the limited effectiveness of most intervention models that are designed in the west, might have worked elsewhere but fail to register the same positive outcomes in sub-Saharan Africa [19–23]. A systematic review of BCI targeting adolescents in SSA was therefore conducted with the objective of delineating this intervention vis-a-vis effectiveness gap and goal of informing future programming.

In terms of organization the review is divided into five major sections some with sub-sections. The first section is the background after which is the methods section. The methods section is further subdivided into three sub-sections: literature search and search period; study eligibility; and study measures. The third section after the methods is the results section. The results section is subdivided into three sub-sections: appraisal of included studies; appraisal of interventions; and appraisal of outcomes which highlights the primary and secondary outcomes targeted by the reviewed studies. After the results section comes the discussion section which analyses the review findings. Lastly there is the conclusion to the review.

## Methods

We conducted this systematic review to identify adolescent BCI strategies relevant to HIV risk and delineate the gaps impeding or facilitating BCI intervention effectiveness in SSA. Seventeen recent interventions implemented in SSA were reviewed. Their effectiveness consistent with targeted outcomes was appraised together with some of their similarities in terms of factors that either facilitate or impede intervention effectiveness. We believe the knowledge gained will help identify flaws that have contributed to the lack of intervention effectiveness relative to behavior change overall in SSA. The knowledge will also help inform the design and modeling of a doctoral intervention by the authors and their team to be developed and implemented in Malawi.

Similar systematic reviews have been conducted in SSA in recent times [6, 9, 13, 14]. The current review differs from most of these reviews on four grounds. First, in that it has incorporated some of the most recent studies not reviewed earlier. Second, it examines in depth factors that either facilitate or impede intervention effectiveness. A majority of previous reviews have focused on only one variable; either factors facilitating intervention success or those impeding success. Third, it is not sorely focused to schools as in Gallant and Maticka-Tyndale or in Paul-Ebhohimhen in view of other contextual and structural dynamics that also factor in to mediate adolescent sexual behaviours but have often been ignored in previous studies [6, 14]. Fourth, contrary to most previous reviews that solely focused on school based interventions, the current intervention has included studies with a community ecological focus not ignoring parental and societal contributions in shaping and determining adolescent behaviour. Reviewed interventions factoring in parental and community dynamics included those by Dancy et al. [24] and Cowan et al. [25]. Finally because it is more up-to-date and most recent compared to other reviews.

### Literature search and search period

From April 2015 to July 2015, a search of literature published from 2000 to 2015 was conducted. Databases searched included PsychINFO, MEDLINE, EBSCOhost, Cochrane, Google Scholar as well as Cambridge and Oxford journal websites, and the UNAIDS and WHO sites. The search strategy included search terms or statements reflecting relevant search algorism that were further qualified for specificity. Outcome measure search terms as; knowledge, attitude, intentions, behaviour, self-efficacy designed to identify programme trials measuring biological, behavioural, cognitive, attitudinal or other outcome not just HIV incidence were used in the search process.

### Study eligibility

Only studies focusing on psychosocial BCI, their effectiveness and implications on adolescent [< 24 years] sexual behaviour relative to the HIV and AIDS pandemic conducted in sub-Saharan Africa were selected for inclusion in this review. Inclusion criteria for review involved assessing psychosocial BCI [non-pharmacological] intended to facilitate risk reduction behaviours relative to HIV and AIDS among adolescents as well as their potential effectiveness. In terms of methodology studies were included if they were experimental or quasi-experimental, specifically randomized and quasi randomized controlled trials. This was done to ensure that evidence was of the highest level of methodological rigour for evaluating programme effectiveness [13, 26]. We assessed methodological quality according to the Cochrane hand book [27]. Articles for review were limited to the period 2000–2015 to filter articles that had been included in many other previous evaluations and thus avoid duplication. Exclusion factors included studies focusing on out of school married adolescents and those that had clinical components in their designs.

Inclusion criteria for the review:

- Peer reviewed publications between 2000 and 2015;
- Focus on psychosocial HIV adolescent BCI strategies conducted in sub-Saharan Africa.

The research methods had to:

- Include experimental or quasi-experimental design or had to be quantitative;
- Had a sample size of at least 100.

Exclusion criteria for the review:

- Publications before 2000;
- Studies which included married adolescents.

### Study measures

Data was extracted first, on the basis of a study prescribing some form of intervention. The characteristics of the interventions more specifically the targeted outcome measures were then evaluated. Studies were included if they explicitly measured changes in knowledge about HIV and AIDS, attitudes or intentions about HIV and AIDS, and sexual behaviours. Other studies that went further to measure psychosocial determinants of sexual behaviours [self-esteem, self-concept, and self -efficacy] were also targeted. Further isolated were those studies that also focused on normative beliefs about abstinence and normative beliefs about as well as reported condom use .

## Results

### Appraisal of included studies

The search yielded 200 titles, in form of full text and abstracts of which 140 were discarded as not relevant or meeting the criteria for adolescent psychosocial BCI studies conducted in sub-Saharan Africa. After further exclusion of studies on grounds of methodology and other inclusion criteria as already highlighted, 17 full-text articles were selected for review. Of the 17 studies, three were randomized controlled trials or community randomized trials [18, 25, 28]. Five of the studies were quasi-experimental [29–33] and the remaining nine were either evaluations, or other studies employing non-experimental methods [6, 9, 14, 24, 34–38]. All the studies were conducted in sub-Saharan Africa with the majority being from southern Africa (*n* = 10) and a few from east Africa (*n* = 3) and the remaining from west Africa (*n* = 4). One study was conducted in South Africa and Trinidad [35] but was included because it included a Southern African country. Most studies focused on high school adolescents with the exception of one that targeted adolescents at a university [37]. A number of studies targeted out of school adolescents (*n* = 5) and had a community intervention element [18, 24, 25, 35, 38]. The age range for respondents in all the studies were from 12 to 24 years as shown in Table i. This is available in Additional file 2: Table S1.

Results of search and study selection are shown on the flow chart in Figure i. This is available in Additional file 1: Figure S1. A total of 17 article with four being captured within evaluations met all criteria and have been reviewed and included in this paper. Characteristics of Studies included for review are shown in Table 1. This is available in Additional file 2: Table S1.

### Appraisal of interventions

Theoretical basis for the interventions were explicit in 10 of the studies [9, 24, 25, 29, 31–35]. The most dominant theory in the intervention frameworks was the Social Cognitive Theory [SCT]. The SCT was used either to inform the content of the intervention or to inform the evaluation or questionnaire design. Some studies alluded to the Health Belief Model and the Theory of Reasoned Action as well as Planned Behaviour [18, 28–30]. Two studies had a community appraisal component in their theoretical framework to inform the study design [31, 35].

All the reviewed studies indicated an active student participatory component in their intervention. Activities ranged from peer awareness and information sharing, class discussions, role modeling, script writing and analysis, drama and community sessions. Peer modeling and psychosocial support activities to change norms and psychosocial determinants of sexual behaviour were the most prevalent strategies in a majority of the studies [9, 24, 29, 32, 33, 37]. A few studies prescribed factual information targeting improvements in knowledge, attitudes and intentions as well as HIV risk reduction within the life skills approach [18, 30, 34].

Programme facilitators included, peer educators trained and supported to implement the programme [24, 29, 32, 33]; trained teachers and trained leaders [9, 18, 28, 30, 38]; teachers and health professionals [34]; national youth service corps and community leaders [31]; identified community leaders and youth leaders [35]. Study messages incorporated facts about transmission, prevention and safe sexual practices; messages about risk reduction, positive norms, as well as psychosocial determinants of sexual behaviour like self-efficacy; and messages targeting normative beliefs about abstinence, condoms and personal risk perception.

Two studies were randomized controlled trials [18, 25] and seven of the studies were quasi experimental including an experimental or intervention arm and a control arm [24, 29–34]. Four studies were contextualized within a rural setting [18, 24, 25, 31]. All of the included evaluations considered major factors that facilitate intervention effectiveness [6, 14, 36].

#### Intervention effectiveness

Intervention effectiveness for this review operationally entails the extent to which interventions successfully registered positive outcomes as intended in their objectives after evaluation. Of the interventions that were evaluated a number registered positive outcomes [25, 28, 29, 32–35]. Some interventions registered success for a particular gender within the cohort for instance boys but not girls [24, 34, 38] or for a particular level of regimen for example full implementation compared to partial implementation [24, 30, 38]. With respect to comparisons based on gender, the Mzake ndi mzake peer group intervention [24] was observed to have had significant benefits on sexual practices and their psychosocial determinants in 16–19 year old male adolescents but not for their 13–15 year old female counterparts. One intervention did not register implementation success overall [9]. Among the factors that were highlighted as facilitating intervention success were: good theoretical basis [31], good programme implementation [25, 28], targeting the right age group [25]. Among the major factors that were highlighted as impeding intervention success were; misaligned theoretical basis and poor implementation [9, 30] as well as targeting the wrong age group of older rather than younger adolescents [9, 18, 30, 34]. The narrow appreciation of SSA issues by model and intervention designers especially from the west was also pointed to as a pitfall to intervention success [29, 31, 33].

### Appraisal of outcomes within the KAPB behavioural model

The KABP behavioural model is integrated as theoretical framework to present the outcome findings in this review. KABP as an HIV behavioural model factors in knowledge, attitudes, practices and behaviours of participants to understand their HIV dynamics. In the current review, assessment of primary and secondary outcome measures was through questionnaires in 10 studies [9, 18, 25, 28–30, 32–34, 37]. Four studies however utilized participatory approaches [24, 31, 35, 38]. These specifically included those employing community participatory strategies in design such as Maticka-Tyndale and the HP4RY Team [31] in Edo state, Nigeria and that by Baptiste et al. [35]. There was heterogeneity in targeted outcome measures. Outcome focus ranged across the continuum from emphasis on only knowledge; a combination of knowledge, attitudes and intentions; to focus on a combination of knowledge, attitudes, behaviour and practices [KABP]. Psychosocial determinants of such sexual practices such as self-esteem, self-concept and self-efficacy were also targeted mostly as secondary outcomes.

#### Knowledge, attitudes and intentions on HIV dynamics - [*n* = 14]

Knowledge regarding HIV and AIDS was evaluated in all the reviewed studies. This was mainly combined with analysis about attitudes and intentions on correlates like abstinence [A], faithfulness [B] and condom use [C]. Intentions on these A, B, + C functions of prevention were also considered by a majority of studies. There were mixed findings regarding the outcomes with changes registered in other interventions as well as gaps at baseline in others. In Cowan et al. [25] for example girls were considered less likely to know about reproductive health issues than boys (*P* < 0.001). In the study by Harvey et al. [28] however, improvements in knowledge (*P* = 0.002) regarding HIV and AIDS were demonstrated in pupils in the intervention arm after 6 months follow up. Attitudes and intentions regarding abstinence as an outcome was reviewed in 12 studies. More significant in these studies, Winskell et al. [38] in a comparative study conducted in 6 SSA countries observed that abstinence was considerably more prominent as a theme. On condoms Agha and van Rossem [29] reported some positive effects with respondents in their intervention arm being more likely to approve of condom use and to intend using condoms immediately after the intervention. Focusing on the B-faithfulness function that is mainly considered with respect to the number of sexual partners, findings were as well mixed. Swartz et al. [32], Magnani et al. [34] and Agha and van Rossem [29] included the outcome in their interventions. Swartz et al. [32] allude to significant reduction in the number of sexual partners in the intervention group at follow up. In the study by Magnani et al. [34] no observed effects on partnering behaviours were registered after the intervention aimed at evaluating the impact of Life skills education on adolescent sexual risk behaviours in Kwazulu-Natal, South Africa.

#### Psychosocial determinants of sexual practices -[*n* = 3]

Psychosocial determinants of sexual practices which include among others self-esteem, self-concept and self-efficacy towards safe sexual practices were not explicitly evaluated in most of the studies except James et al. [30], Swartz et al. [32] and Magnani et al. [34]. In James et al. [30] no effects were found on the outcome measure after the intervention. Swartz et al. [32] registered significant improvements of psychosocial skills following the intervention. In the Magnani et al. [34] study significant effects were observed on selected areas of sexual-reproductive health and behaviours more prominent being in self-efficacy.

#### Sexual behaviour - [*n* = 10]

Sexual behaviours incorporating sexual experience, safer sexual practices using condoms, concurrence and multiple partnerships were evaluated in a majority of studies with mixed results as well. In Visser [33] the percentage of learners in the experimental group who were sexually experienced remained unchanged over the duration of the 18 months slated for the intervention. In the control group however, a significantly increased percentage of learners were sexually experienced after the same time. In the study by Harvey et al. [28] for schools receiving the drama programme, sexually active pupils reported an increase in safe sexual behaviours especially condom use. In the study by Dancy et al. [24] compared to their counterparts in the control community, the adolescents in the Mzake ndi mzake kuunikira achinyamata [MMKA] community had significantly better scores on the outcome variables on safer sexual behaviours and the psychosocial determinants of such including attitude towards condom use and condom self-efficacy. In Agha and van Rossem [29] significant reduction in unsafe sexual behaviours specifically multiple sexual partnerships were reported. Contrary to the significant effects registered in the aforementioned studies, in the study by James et al. [30] however, no effects were found on safer sex practices (condom use and number of sexual partners) or on psychosocial determinants of these practices. Similarly for Magnani et al. [34] no consistent effects were observed on age at sexual initiation, secondary abstinence, or partnering behaviours following the intervention.

## Discussion

We have presented findings from a systematic review on the literature on BCI among adolescents in SSA. The findings were discussed within the KABP behavioural theoretical framework. We observed that there are diverse factors that limit the effectiveness of programmes ranging from; misaligned theoretical framework, implementation bottlenecks, misdirected targeting of primary beneficiaries, narrow focus on individual determinants of sexual behaviour and the limited effectiveness of most intervention models that are designed in the west and are not feasible and appropriate in sub-Saharan African contexts. Research evidence however suggests that the efficacy and effectiveness of BCI is grounded on specific characteristics that typify most successful interventions [12]. Failure by most programmes in sub-Saharan Africa to register their intended outcomes among adolescents comes against a background of high rates of new HIV infections among adolescents in most countries in the region. This gap remains a cause for concern among public health experts and AIDS specialists. An assessment of some of the factors derailing programmatic success in the current review was therefore necessary to guide and inform future programming in the region.

Michielsen [9] and others [6, 11, 12] point to misaligned theoretical framework as one factor that derails BCI programme effectiveness. Several reviewed studies based their designs on behavioural theories. These theories which include the Health Belief Model [HBM], Social Learning or Social Cognitive Theory [SCT], and the Theory of Reasoned Action or Planned Behavior [TRA] mostly focus on cognitions as the foundation of sexual behaviour. These theories have often been criticized for being too simplistic and reductionist in their analysis of sexual behaviour [14, 36]. It is argued that sexual behaviour is far too complex a phenomena transcending individual dynamics [39–42] and that that sex is determined by multiple factors. Rather than being intra-personally determined for example, Michielsen [9] in one of the reviewed studies conducted in Rwanda identifies such other variables as; interpersonal, environmental, social- cultural and structural factors like gender, societal norms and poverty as determining sexual behaviour among adolescents. Cognitive behavioural theories have also been deemed limited in that they predominantly focus on individual determinants of sexual behaviour at the expense of the socio-cultural and structural variables [9, 41]. By ignoring socio-cultural variables, such interventions that target individuals determinants have also been empirically proven to only have efficacy in changing behaviour over the short-term. Gaps in the sustainability and transferability of those short-term changes in ‘real world’ scenarios have led to criticisms concerning the applicability of the theories to HIV prevention. Considering these criticisms what is needed are theoretical models that consider sexual practices as diverse, contextual, historyed and partnered [39, 43]. Others have recommended shifting intervention efforts toward multi-level strategies that include not only the individual but also community and societal initiatives [41, 42, 44, 45]. Maticka-Tyndale and the HP4RY Team [31] in one of the reviewed studies for instance set their intervention within the multi-level Ecological framework and specifically used sexual scripting theory and the AIDS Competent Community theoretical framework.

Apart from the theoretical pitfalls, BCI interventions targeting adolescents have also been proven to lack effectiveness in sub-Saharan Africa because they fail to target the right age category albeit with the right type of intervention. That is an age group that might practically have greater odds of benefiting from that type of intervention - whether the intervention is targeting risk avoidance or risk reduction. In our case in the current review, a majority of interventions generally targeted late adolescence −15-24 years [18, 25, 28–30, 32, 34, 37].

Evidence from previous research indicates that interventions that generally target pre or early adolescence, whatever the focal outcome; register more positive outcomes than those targeting late adolescence. Targeting pre or early adolescence is justified by the likelihood of a majority of such young people not having initiated risky sexual behaviours. There is therefore the probability of such younger adolescents benefiting more from intervention objectives. In Harvey et al. [28] in our current review for instance the intervention was not able to affect a change in sexual behaviours in the 17–18 years age group [e.g., abstinence and number of sexual partners] although the program was able to increase condom use. Further empirical evidence points to the feasibility of better chances to establish low-risk behaviours than to change existing behaviours resulting in calls to target younger adolescents [15–17]. This however does not imply that interventions for older adolescents be discontinued. Rather the observation calls for tailoring or realigning interventions by designing them differently in tandem with the age categories of adolescents, whether early or late. Interventions for younger adolescents on the one hand could for example target risk avoidance skills mostly abstinence and delayed sexual debut taking cognizance of the fact that most of them may still be sexually inactive. On the other hand, realizing that older adolescents are more likely to be sexually active and hence less likely to benefit from such skills as abstinence or delayed debut although they are the highest risk group for new infections it would be prudent to realign intervention focus. Interventions targeting older adolescents therefore need to be tailored and could best focus on risk reduction skills such as faithfulness to one partner [zero-grazing] and condom use if they are to effectively register positive outcomes.

Lack of intervention effectiveness has also been ascribed to implementation bottlenecks. Most evaluations in the current review have pointed to implementation flaws as the major factor limiting programmatic success in the fight against HIV and AIDS among young people in sub-Saharan Africa. Whether reviewers seek to avoid blaming western models or the nature of their design. Whether they focus on the plausible relevance or applicability in a different context with differing norms, contextual or structural dynamics stands to be validated. Green and Witte [46] point to complications and influences imparted on the design and implementation of AIDS programmes by the sexually related values, beliefs, and attitudes of western - especially American researchers. Such experts often design and implement programmes without regard to traditional African values and socio-cultural norms. With most interventions designed in the west sometimes without proper piloting; implementation therefore becomes a major limiting factor, either through design and planning pitfalls at the outset [13, 24, 31] or other multiple variables. Such other variables may include structural variables as poverty or gender disparities [10, 46, 47, 48]; or even contextual complexities like teacher attrition [11, 17, 20], resource scarcity in public schools or teacher and community reluctance to endorse certain programme requirements such as the condom [19, 20, 35]. Some interventions however seem to have registered success amidst the plethora of challenges. In the current review, interventions prescribing peer education seem to have registered positive outcomes [24, 29, 32, 33, 37]. According to other previous reviews [6, 31, 40, 41] peer education programmes place individuals and their actions [e.g., sexual practices] within the context of social relationships such as families, partnerships and peer groups. Peer-led programmes typically target groups and communities rather than individuals as the unit of change, with agents of change coming from within the group or community [i.e., peers] rather than brought from outside. Peer education is based on the premise that ‘if enough agents of change within the population visibly adopt, endorse, and support change in behaviour’, if the association of influences be functionally high between peers; then norms and behaviours are most likely to change because they are influenced by significant others who are liked and trusted group members [40, 41, 49–53]. The element of trust and involvement is vital in peer education and might have been the potential variable culminating in the approach’s success relative to other programmes reviewed.

The same gap still re-echoes in HIV and AIDS research with respect to outcomes in this review. A majority of interventions registered positive outcomes on knowledge and attitudes about HIV and AIDS. When it came to translating those improvements into actual behaviour a gap was however identified [9, 24, 28, 30, 34]. Interventions have to a large extent registered improvements with respect to knowledge but that knowledge has not necessarily translated to sexual behavioural change. There are so many variables that derail behavioural change in sub-Saharan Africa ranging from socio-cultural, socio-economic and other structural dynamics as poverty and gender disparities [3, 5, 10, 41, 44, 45, 48, 54]. This has tempted the authors to hypothesize that the problem of surmounting the AIDS pandemic in sub-Saharan Africa is not a problem of lack of intervention efficacy or effectiveness. Rather it is a problem that can best be attributed to the myriads of cultural complexities that often interact to derail intervention effort. There are a plethora of diverse cultural myths, negative social-constructions, misconceptions and rumors that surround AIDS issues in sub-Saharan Africa. These cultural relics have been demonstrated to be strong barriers to intervention effort [1, 5, 47, 48, 55]. Intervention programmes therefore have to be designed and developed with such socio-cultural complexities in perspective.

## Conclusion

Our systematic review exposes the dearth and paucity in psychosocial BCI studies targeting adolescents in SSA. There are however mixed findings about the effectiveness of such BCI interventions targeting adolescents. Among the major noted factors derailing intervention effectiveness are; misaligned theoretical focus, implementation bottlenecks, targeting the wrong age group especially older instead of younger adolescents and pitfalls associated with model design with most interventions designed in the west not factoring in SSA cultural dynamics and other structural factors. In terms of outcomes, the lack of a link between knowledge and change in behaviour still re-echoes in SSA HIV and AIDS research. Among the interventions that registered positive outcomes peer education stands out as being more efficacious and effective in facilitating HIV risk reduction. There is therefore need for more studies on BCI targeting adolescents in SSA and interventions employing peer education need to be studied further to determine reasons why they are registering outcome successes. This will help inform future programming in the area of adolescent HIV and AIDS prevention.

## Additional files

Additional file 1: Figure S1. Flow chart for study selection (DOCX 17 kb)

Additional file 2: Table S1. Table showing characteristics of studies included (DOCX 16 kb)

## Abbreviations

AIDS: Acquired immunodeficiency syndrome
BCI: Behaviour change interventions
HIV: Human immunodeficiency virus
KAPB: Knowledge, attitudes, practices and behaviour
NIH: National institute of health
SSA: sub-Saharan Africa
