# Supplementary material for: Systematic review: a review of adolescent behavior change interventions [BCI] and their effectiveness in HIV and AIDS prevention in sub-Saharan Africa
Source: BMC Public Health. 2017 Sep 18;17:718. doi: 10.1186/s12889-017-4729-2 (PMC5604191; doi:10.1186/s12889-017-4729-2)
Supplement: Supplementary file 2 — Table showing characteristics of studies included (DOCX 16 kb) [file 12889_2017_4729_MOESM2_ESM.docx]

**Table 1**: Characteristics of studies included in systematic review.

| Author | Yr of publication | Title | Journal | Sex and Age range | Study design | Category/Classification of intervention | Remarks |
| --- | --- | --- | --- | --- | --- | --- | --- |
| Agha S and van Rossem | 2004 | Impact of a school-based peer sexual health intervention on normative beliefs, risk perception and sexual behaviour of Zambian adolescents  **[Zambia]** | Journal of Adolescent Health 34: 441-452 | Male and Female [14 - 23 years] | Quasi-experimental | Peer education | A single session school-based peer sexual health intervention resulted in the development of normative beliefs about abstinence that were sustained over a 6-month period. |
| Baptiste DR, Bhana EA et al | 2006 | Community collaboration youth-focused HIV/AIDS prevention in South Africa and Trinidad **[South Africa]** | Journal of Pediatric Psychology 31(9): 905-916 | Male and Female [ 11 - 13 years] | Community participatory approach | Community collaboration | High recruitment and retention in community participation with youth talking more about sensitive issues on HIV/AIDS were observed. |
| Cowan FM, Pascoe SJS, Langhaug LF et al | 2008 | The Regai Dzive Shiri Project: a cluster randomized controlled trial to determine the effectiveness of a multi-component community-based HIV prevention intervention for rural youth in Zimbabwe-study design and baseline results  **[Zimbabwe]** | Tropical Medicine and International Health 13(10): 1235-1244 | Male and Female [ 13 - 19 years] | RCT | Community peer intervention | Low rates of HIV after evaluation suggest that the intervention was started before the population became sexually active indicating that targeting the very young may boost programme outcome effectiveness. |
| Dancy BL, Jere DL et al | 2014 | HIV risk reduction intervention for rural adolescents in Malawi  **[Malawi]** | Journal of HIV/AIDS Social Services 13(3): 271-291 | Male and Female [ 13 - 19 years] | Quasi-experimental | Peer education [Mzake ndi mzake kuunikira achinyamata) | The intervention had significant benefits for male and 16-19 year old adolescents, but not for 13-15 year old female adolescents. |
| Gallant M and Maticka-Tyndale E | 2004 | School-based HIV prevention programmes for African youth  **[SSA]** | Social Science & Medicine 58: 1337-1351 | All genders | Quasi-experimental with pre-post test assessments | Evaluation | Knowledge and attitudes are easiest to change but behaviours are much more challenging although more research is needed to identify, with certainty, the factors that drive successful school-based HIV/AIDS risk reduction programs. |
| Harvey B, Stuart J and Swan T | 2000 | Evaluation of a drama-in-education programme to increase AIDS awareness in South African high schools  **[South Africa]** | Journal of Sexually transmitted diseases and AIDS 11(2): 105-111 | Male and Female [14 - 19 years] | Community Randomized Intervention Trial [CRIT] | Drama-in- education | Drama was seen as being effective as an intervention in spreading awareness about HIV and AIDS. |
| James S, Reddy P et al | 2006 | The impact of an HIV and AIDS Life skills program on secondary school students in Kwazulu-Natal, RSA  **[South Africa]** | AIDS Education Preview 18(4): 281-294 | Male and Female [12 -19 years] | Quasi- experimental | Life skills | An exploratory analysis showed that students who received the full Life skills intervention were more positive in their perceptions about sexual behaviour. |
| Jewkes R, Nduna M et al | 2006 | A cluster randomized controlled trial to determine the effectiveness of Stepping Stones in preventing HIV infections and promoting safer sexual behavior amongst youth in the rural Eastern Cape, South Africa: trial design, methods and  baseline findings.  **[South Africa]** | Tropical Medicine and International Health 11(1): 3-16 | Male and Female [ 13 - 19 years] | RCT | Stepping Stones Life Skills | This was the third RCT to be conducted in sub-Saharan Africa evaluating a behavioural intervention using HIV incidence as a primary outcome and there was good baseline comparability between the study arms and the process data suggesting feasibility and adequate implementation. |
| Kirby DB | 2008 | The impact of Abstinence and Comprehensive Sex and STD/HIV Education Programs on Adolescent Sexual Behaviour | Sexuality Research & Social Policy 5(3): 18 - 27 | All genders | Quasi-experimental & RCT studies | Evaluation | Based on the review, abstinence programs have little evidence to warrant their widespread replication, and conversely, strong evidence suggests that some comprehensive programs should be disseminated widely. |
| Magnani R, Macintyre K et al | 2005 | The impact of Life skills education on adolescent sexual risk behaviours in Kwazulu-Natal  **[South Africa]** | Journal of Adolescent Health 36(4): 289-304 | Male and Female [14 - 24 years] | Dose-response appraisal & econometric simulation | Life skills | School-based life skills education appears capable of communicating key information and helping youth develop skills relevant to reduce HIV risk. |
| Maticka-Tyndale E and the HP4RY Team | 2012 | Bridging Theory and Practice in HIV Prevention for Rural Youth, Nigeria  **[Nigeria]** | African Journal of Reproductive Health 16(2): 39-53 | Male and Female [14 - 20 years] | Quasi-experimental | Sexual scripting | HP4RY as a set within the multi-level Ecological framework specifically using sexual scripting theory is effective in guiding action research on HIV/AIDS in Junior secondary schools. |
| Michielsen K | 2012 | HIV prevention for young people in Sub-Saharan Africa: effectiveness of interventions and areas for improvement. Evidence from Rwanda  **[Rwanda]** | Africa Focus 25(2): 132-146 | Male and Female [15 - 24 years] | NRCT | Peer education | The limited effectiveness of the peer education programme intervention in Rwanda paralled the problems identified in the literature review and meta-analysis for the study. |
| Othero DM, Aduma P and Opil CO | 2009 | Knowledge, attitudes and sexual practices of University students for advancing peer HIV education  **[Kenya]** | East African Medical Journal 86(1): 11-15 | Male and Female [15 - 24 years] | Cross sectional study to facilitate development and implementation of a peer education programme | Peer education | Peer influence emerged as an important feature in accelerating risky sexual behaviour hence the need for advancing peer education. |
| Paul-Ebhohimhen VA, Poobalan A and van Teijlingen ER | 2008 | A systematic review of school-based sexual health interventions to prevent STI/HIV in sub-Saharan Africa  **[SSA]** | BMC Public Health 8(4): 1-13 | All genders | Quasi-experimental and RCT studies | Evaluation | There is a great need in sub-Saharan Africa for well-evaluated and effective school-based sexual health interventions. |
| Swart S, Deutstsch C et al | 2012 | Measuring change in vulnerable adolescents: findings from a peer education evaluation in South Africa  **[South Africa]** | SAHARA Journal 9(4): 242-254 | Male and Female [14 - 16 years] | Quasi- experimental | Peer education | Peer education programmes provide vulnerable youth with opportunities to develop psychosocial skills and informational resources that contribute to the changing of norms, attitudes and behaviours. |
| Visser MJ | 2007 | HIV/AIDS prevention through peer education and support in South Africa  **[South Africa]** | SAHARA Journal 4(3): 678-694 | Male and Female [13 - 20 years] | Quasi- experimental | Peer education | Peer education can contribute to a delayed onset of sexual activity, and can therefore contribute to the prevention of HIV and AIDS amongst adolescents. |
| Winskell K, Beres LK et al | 2011 | Making sense of abstinence: social representations in young Africans' HIV-related narratives from six countries  **[SSA]** | Culture, Health & Sexuality | Male and Female [10- 24 years] | Thematic-analysis | Narrative Scripting | Examples of non-stigmatising pro-abstinence messaging highlighted the appeal of discourses of romantic love and future plans across countries and demographic characteristics. |
